# Association of effective dose to immune cells and vertebral marrow dose with hematologic toxicity during neoadjuvant chemoradiotherapy in esophageal squamous cell carcinoma

**DOI:** 10.1186/s12885-024-12531-z

**Published:** 2024-06-28

**Authors:** Meng Zhang, Zhenjiang Li, Yong Yin

**Affiliations:** 1 Shandong University Cancer Center, Shandong University, Jinan, Shandong China; 2 Department of Radiation Oncology Physics and Technology, Shandong Cancer Hospital and Institute, Shandong First Medical University, Shandong Academy of Medical Sciences, Jinan, Shandong China

**Keywords:** Esophageal squamous cell carcinoma, Neoadjuvant chemoradiotherapy, Hematologic toxicity, Dosimetry parameters

## Abstract

**Background:**

To explore the correlation between effective dose to immune cells (EDIC) and vertebral bone marrow dose and hematologic toxicity (HT) for esophageal squamous cell carcinoma (ESCC) during neoadjuvant chemoradiotherapy (nCRT).

**Methods:**

The study included 106 ESCC patients treated with nCRT. We collected dosimetric parameters, including vertebral body volumes receiving 10–40 Gy (V10, V20, V30, V40) and EDIC and complete blood counts. Associations of the cell nadir and dosimetric parameters were examined by linear and logistic regression analysis. The receiver operating characteristic (ROC) curves were used to determine the cutoff values for the dosimetric parameters.

**Results:**

During nCRT, the incidence of grade 3–4 lymphopenia, leukopenia, and neutropenia was 76.4%, 37.3%, and 37.3%, respectively. Patients with EDIC ≤ 4.63 Gy plus V10 ≤ 140.3 ml were strongly associated with lower risk of grade 3–4 lymphopenia (OR, 0.050; *P* < 0.001), and patients with EDIC ≤ 4.53 Gy plus V10 ≤ 100.9 ml were strongly associated with lower risk of grade 3–4 leukopenia (OR, 0.177; *P* = 0.011), and patients with EDIC ≤ 5.79 Gy were strongly associated with lower risk of grade 3–4 neutropenia (OR, 0.401; *P* = 0.031). Kaplan-Meier analysis showed that there was a significant difference among all groups for grade 3–4 lymphopenia, leukopenia, and neutropenia (*P* < 0.05).

**Conclusion:**

The dose of vertebral bone marrow irradiation and EDIC were significantly correlated with grade 3–4 leukopenia and lymphopenia, and EDIC was significantly correlated with grade 3–4 neutropenia. Reducing vertebral bone marrow irradiation and EDIC effectively reduce the incidence of HT.

## Introduction

Esophageal squamous cell carcinoma (ESCC) is a common digestive system malignancy worldwide. It is the seventh most common malignancy globally [1, 2]. At present, neoadjuvant chemoradiotherapy (nCRT) is the standard treatment for locally advanced resectable esophageal cancer. The evidence suggests that nCRT plus surgery improves overall survival compared with surgery alone among patients with locally advanced ESCC [3, 4]. However, severe acute hematological toxicity (HT) often occurs during nCRT and is associated with poor outcomes [5]. The presence of myelosuppression during treatment can also affect the progress of the treatment plan, leading to dose reductions and interruptions of radiotherapy [6]. Several studies have found that increased radiation doses in bone marrow during nCRT are associated with acute HT in a variety of cancer, including esophageal cancer [7–10]. In addition to the bone marrow, increased radiation doses to the heart, lung, and blood vessels, which were the organs with large blood supplies, may also produce a decrease in peripheral blood leukocytes leading to immunosuppression [11]. The innate and adaptive immune system plays a significant role in the progression of tumor and patient prognosis in cases of solid malignancies. Tumor-associated inflammation, mostly maintained by innate immune cells, promotes tumor growth in various contexts. Inflammatory, regenerative, and anti-inflammatory cytokines are released in response to initial innate activation, which in turn influences how the adaptive immune system reacts to the tumor [12]. Lymphocytes are a crucial part of cell-mediated immunity systems and are highly radiosensitive. They can be destroyed by radiotherapy even at very low radiation doses of < 1 Gy [13]. Jin and colleagues developed a model to estimate effective dose to immune cell (EDIC) to substitute the estimated dose of circulation immune pool, such as the heart, lung, blood vessels, and other organs [14]. Cai et al. found that higher EDIC was correlated with severe radiation induced lymphopenia and poorer clinical outcomes in patients with ESCC [15]. Tseng et al [16] found the lower radiation dose to the spleen and bone marrow plus the lower EDIC reduced the risk of grade 4 lymphopenia during definitive concurrent chemoradiotherapy in ESCC. Neutrophils could promote tumor angiogenesis, invasion, and metastasis to facilitate tumor progression primarily. Meanwhile, neutrophils can establish a bidirectional interaction with platelets, lymphocytes, and other components that can influence cancer outcomes [17, 18]. Accumulating evidence has found that high levels of neutrophils in blood and high neutrophil-to-lymphocyte ratios are associated with a poor prognosis in cancer [19].

Therefore, we developed this study to explore the correlation between EDIC and vertebral marrow dose and HT, which could provide possibilities for optimizing a radiotherapy plan to reduce the incidence of HT.

## Materials and methods

### Patients and treatment

We identified 106 patients with clinical stage II-IVa ESCC (according to the eighth edition of the American Joint Committee on Cancer staging system) treated at Shandong Cancer Hospital with nCRT between May 2017 and May 2021. The institutional review board approved our study. We included patients if they were pathologically confirmed ESCC, completed nCRT followed by esophagectomy, had at least four available complete blood counts (CBC) records during nCRT weekly, and had available dosimetric parameters. Patients were excluded if they had distant metastatic disease, received a radiation dose less than 40 Gy or more than 50 Gy, had a history of radiotherapy, and had chronic inflammatory or autoimmune disease. Patients were treated with the intensity-modulated radiation therapy (IMRT) technique, most at a prescription dose of 41.4 Gy in 23 daily fractions, and a few at 40 Gy in 20 daily fractions or 45 Gy in 25 daily fractions, and concurrent or sequential cisplatin/taxane (TP) chemotherapy regimen (weekly paclitaxel 50 mg/m^2^ or docetaxel 30 mg/m^2^ and cisplatin 25 mg/m^2^ or triweekly paclitaxel 175mg/m^2^ or docetaxel 75 mg/m^2^ and cisplatin 75 mg/m^2^) or cisplatin/fluorouracil (PF) chemotherapy regimen (triweekly fluorouracil 1000mg/m^2^ and cisplatin 75 mg/m^2^). And patients who received induction chemotherapy did not have normal CBC at the start of nCRT and developed grade ≥ 2 HT according to the Common Terminology Criteria for Adverse Events (CTCAE) version 5.0 during the induction chemotherapy were excluded. Esophagectomy was performed within 4 to 8 weeks after the completion of nCRT.

### Data collection

We retrospectively contoured the vertebral body bone marrow on radiation therapy (RT) planning CT scans, whose superior and inferior borders depend on the isodose line of PTV. The vertebral body volumes (ml) receiving 10–40 Gy (V10, V20, V30, V40) were recorded from the dose volume histogram (DVH) from Eclipse treatment planning systems. And the following parameters were also collected: RT technique, radiation dose, gross tumor volume (GTV), mean heart dose (MHD), mean lung dose (MLD), mean liver dose (MlD), mean body dose (MBD), and integral total dose volume (ITDV). In addition, some clinical factors were also collected, including age, gender, smoking history, alcohol history, tumor location, clinical stage, and CBC, including white blood cell count (WBC), neutrophil count (NEU), absolute lymphocyte count (ALC), platelet count (PLT), and hemoglobin (HGB) before and during (weekly) nCRT. The cell nadir was defined as the lowest cell count during nCRT. HT were graded according to the CTCAE version 5.0.

### EDIC calculation

The EDIC model which was constructed by Jin et al [14] was assumed that radiation dose was uniformly delivered to all cells for rapidly circulating ones, including the heart, lung, and blood vessels, and only to those in the irradiated volume for slowly circulating ones, like the lymphatic system and blood reservoirs (a portion of veins/capillaries). The equivalent uniform dose (EUD) to the total blood was calculated from the differential DVH that were computed using a similar model reported by Yovino S et al [20]. The EDIC was considered the sum of EUDs of organs that are irradiated in esophageal nCRT, mainly including lung, heart, liver, large vessels, and small vessels and capillaries in other organs. (estimated percentage of cardiac output: 12%, 8%, 15%, 45%, and 35%, respectively) [21]. The EUD of lung, heart, and large vessels was calculated as the percentage of cardiac output multiply mean organ dose (MOD). Integral total body dose can be used to replace the MOD for the large vessels and small vessels and capillaries in other organs, and 0.85 is a dose effectiveness factor for liver, small vessels, and capillaries. Therefore, the EDIC was calculated as follows [21]:

$$\begin{array}{l}EDIC = 0.12*MLD + 0.08*MHD + 0.15*0.85*{\left( {\frac{n}{{45}}} \right)^{\frac{1}{2}}}\\ *MlD + [0.45 + 0.35*0.85*{\left( {\frac{n}{{45}}} \right)^{\frac{1}{2}}}]*ITDV/\left( {61.8*{{10}^3}} \right)\end{array}$$

Since the percentage of large and small vessels were for the entire body, and CT images did not include the entire body. Therefore, we used the Vscan/61.8 × 10^3^ factor to adjust the percentage of large and small vessels, which was achieved by assuming that each patient weighed 140 ponds, or 63 kg, or had a volume of 61.8 × 10^3^ CC [21].

### Statistical analysis

We used univariate linear regression analysis to examine the correlation between the dosimetry parameters along with clinicopathologic features and cell nadir. When multiple one marrow dosimetric parameters showed significance on univariate, we incorporated the variable with the lowest univariate *p*-value for multivariate analysis. We used Benjamini-Hochberg false discovery rate (FDR) correction for multiple hypothesis testing. Adjusted *P* values are denoted as Q values. To avoid multicollinearity, we used receiver operating characteristic curve (ROC) to transform the dosimetry parameters into binomial variables with the endpoint being the occurrence of grade 3 cell toxicity. Based on the cutoff values of the dosimetry parameters, patients were divided into subgroups, including the “all lower” group (patients who received EDIC and vertebral body volumes ≤ their corresponding best cutoffs), the “all higher” group (patients who received EDIC and vertebral body volumes > their corresponding best cutoffs), and the remaining patients were included in the “others” group. We used the Kaplan-Meier method to analyze the comparison of the cumulative incidence of cell toxicity between the dosimetry subgroups. All analyses were performed using SPSS (Version 26.0, IBM) and GraphPad Prism 8.0 software. Significance was defined as variables with a two-sided *P* value < 0.05.

## Results

### Patients characteristics

There were 106 patients incorporated in this study with a median age of 61 years (ranging from 48 to 73). Most patients were male (84.9%), with stage II-IVa disease (74.5%). There were 53 (50.0%) patients with tumors in the distal segment of the esophagus and others in the middle segment of the esophagus and above. Most patients received a radiation dose of 41.4 Gy (83.0%) and were treated with TP (89.0%) chemotherapy regimen. These results are summarized in Table 1.

**Table 1:** Patient characteristics

Characteristic	Number of Patients (%)
**Sex**	
Male	90 (84.9%)
Female	16 (15.1%)
**Age**	
≤ 60 years	39 (36.8%)
> 60 years	67 (63.2%)
**Smoking history**	
No	48 (45.3%)
Yes	58 (54.7%)
**Alcohol history**	
No	52 (49.1%)
Yes	54 (50.9%)
**Tumor location**	
Upper	53 (50.0%)
Middle + Distal	53 (50.0%)
**GTV**	
≤ 48.4cm^3^	53 (50.0%)
> 48.4cm^3^	53 (50.0%)
**Clinical stage**	
II	27 (25.5%)
III-IVa	79 (74.5%)
**Radiotherapy dose**	
≤ 41.4 Gy	94 (88.7%)
> 41.4 Gy	12 (11.3%)
**Chemotherapy regimen**	
FP	17 (16.0%)
TP	89 (84.0%)

GTV, gross tumor volume; PF cisplatin/fluorouracil; TP cisplatin/taxane

### CBC data

**Fig. 1:**
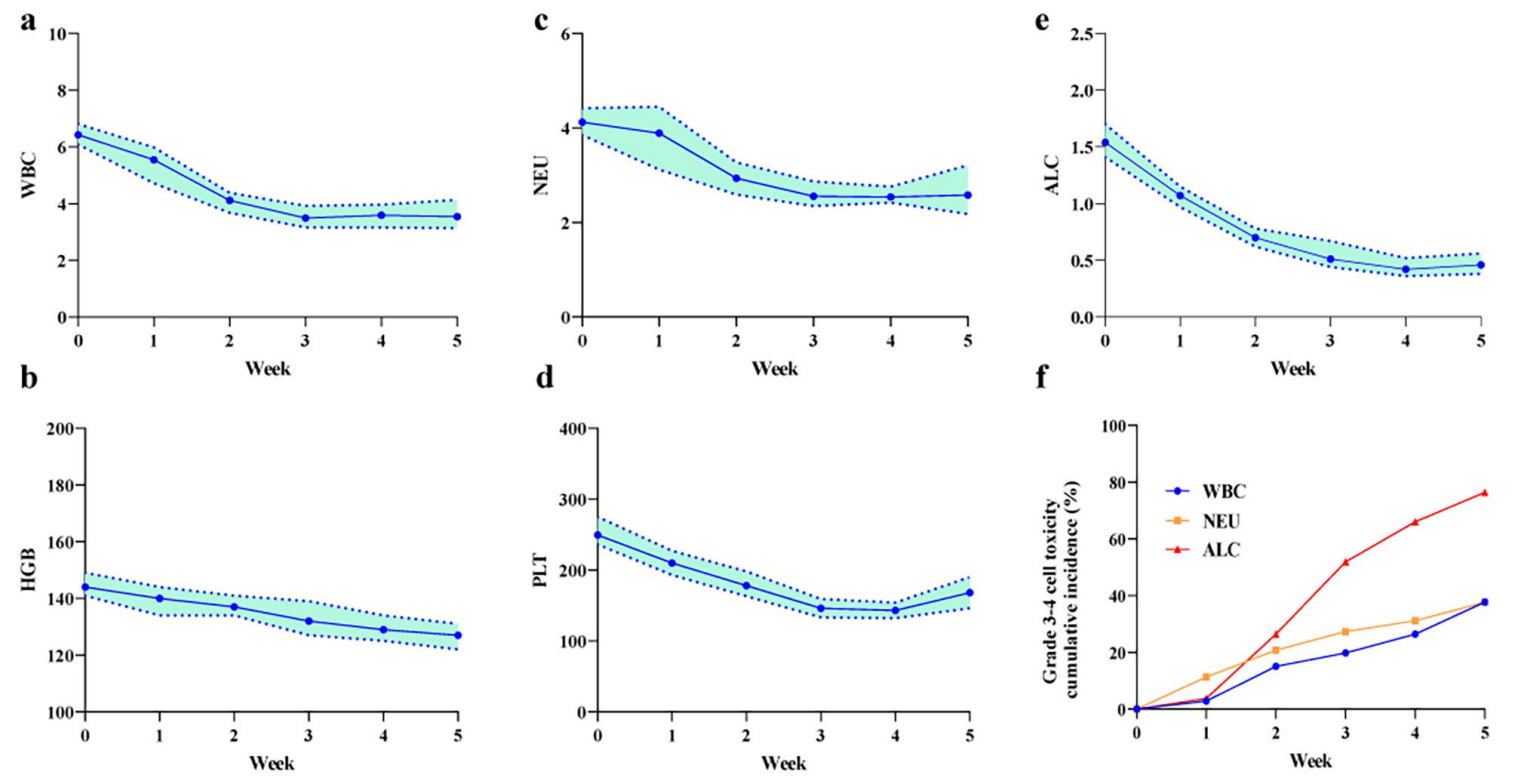
Hematologic cell trends (**a-e**) and grade 3–4 cell toxicity cumulative incidence (**f**) during nCRT for esophageal squamous cell carcinoma. ALC absolute lymphocyte count; WBC white blood cell count; NEU neutrophil count; PLT platelet count; HGB hemoglobin

We acquired 106 patients’ CBC data, including WBC, NEU, ALC, HGB, and PLT from baseline until nCRT completion. The details of the CBC data are shown in Fig. 1. WBC, NEU, and ALC declined per week throughout nCRT with the rapid declines in the first two weeks, and generally reached nadir at week 3–4, and then recovered and continued to stabilize. HGB declined continuously from the beginning untill the end of nCRT. PLT steadily declined per week and reached nadir at week 3–4, and then quickly recovered.Meanwhile, the cell counts for week 1–5 during nCRT were as follows. For WBC, the median of baseline was 6.43 × 10^9^/L, and it declined to 5.55, 4.10, 3.49, 3.59, and 3.54 × 10^9^/L, respectively. For NEU, the median of baseline was 4.12 × 10^9^/L, and it declined to 3.89, 2.94, 2.56, 2.54, and 2.58 × 10^9^/L, respectively. For ALC, the median of baseline was 1.54 × 10^9^/L, and it declined to 1.07, 0.70, 0.51, 0.42, and 0.46 × 10^9^/L, respectively. For HGB, the median of baseline was 144 g/L, and it declined to 140, 137, 132, 129, and 127 g/L, respectively. For PLT, the median of baseline was 249.5 × 10^9^/L, and it declined to 210, 178, 146, 143, and 168 × 10^9^/L, respectively. During CRT, 81 (76.4%) developed at grade 3–4 lymphopenia, 40 patients (37.3%) developed grade 3–4 leukopenia, 40 patients (37.3%) developed grade 3–4 neutropenia, only 1 patients (0.94%) developed grade 3–4 anaemia, and no patients developed grade 3–4 thrombocytopenia.

### Dosimetric parameters associated with HT

**Fig. 2:**
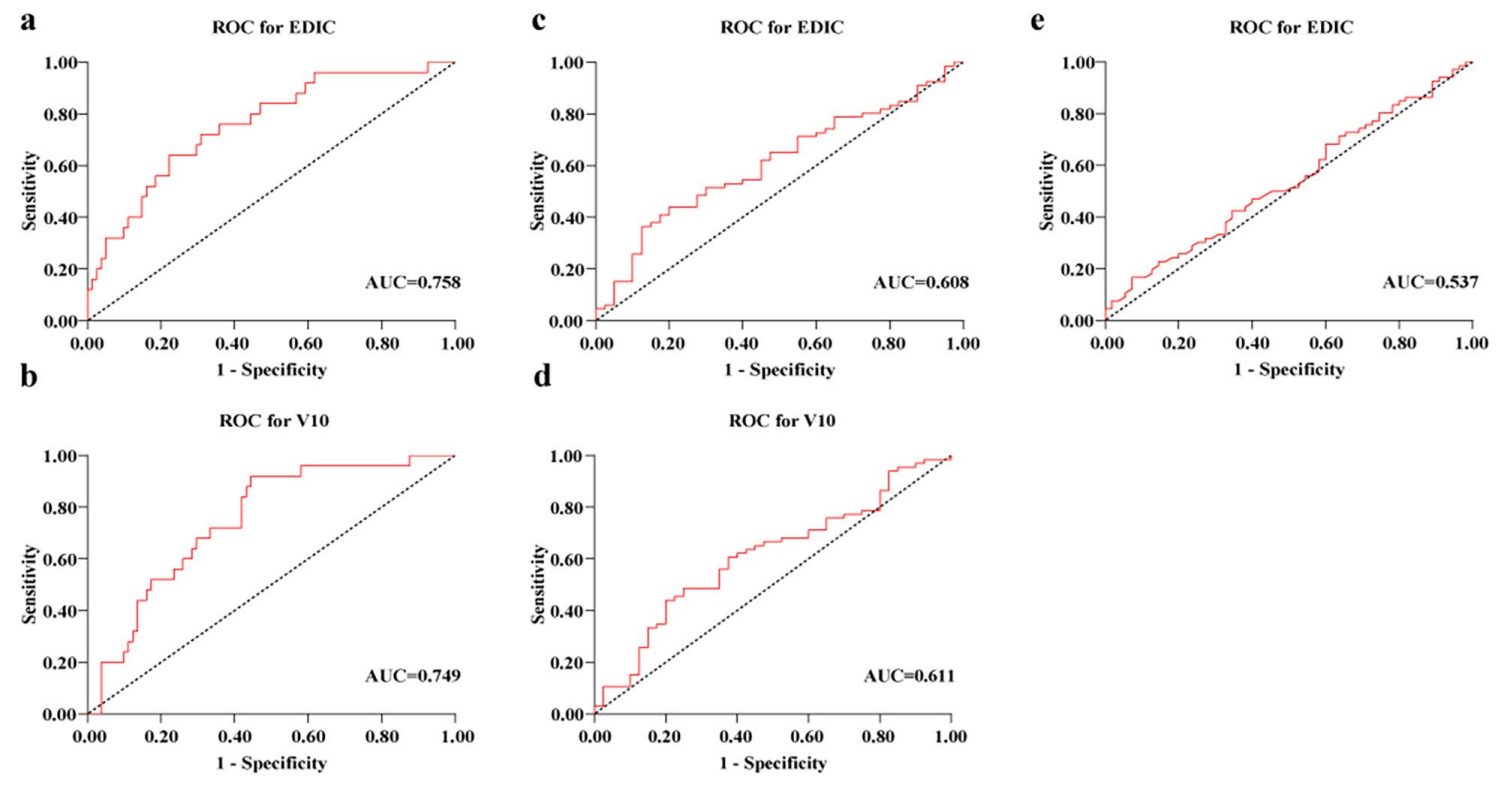
ROC curves for the dosimetry parameters with the endpoint of grade 3–4 cell toxicity. ROC curves for EDIC **(a)** and V10 **(b)** with the endpoint of grade 3–4 lymphopenia; ROC curves for EDIC **(c)** and V10 **(d)** with the endpoint of grade 3–4 leukopenia; ROC curves for EDIC **(e)** with the endpoint of grade 3–4 neutropenia. ROC receiver operating characteristic; EDIC effective dose to immune cells

We performed univariate linear regression analysis to identify associations between clinicopathologic features of age, sex, smoking history, alcohol history, tumor location, GTV, clinical stage, chemotherapy regimen, and dosimetric parameters with cell count nadirs. These results are displayed in Table 2. For WBC, EDIC (*P* < 0.004), V10 (*P* < 0.008), V20 (*P* < 0.015), and age (*P* < 0.041) were associated with leukopenia. For NEU, EDIC (*P* < 0.022) and chemotherapy regimen (*P* = 0.040) were associated with neutropenia. For ALC, EDIC (*P* < 0.001), V10 (*P* < 0.001), V20 (*P* < 0.001), V30 (*P* < 0.001), V40 (*P* < 0.001), and tumor location (*P* = 0.037) were associated with lymphopenia. ROC curves were then made for the significient dosimetry parameters with the endpoint of grade 3 HT to obtain the cutoff values (Fig. 2). For ALC, EDIC < 4.63 Gy plus V10 < 140.3 ml went into “all lower” group, and EDIC ≥ 4.63 Gy plus V10 ≥ 140.3 ml went into “all higher” group, and remaining went into “others” group; for WBC, EDIC < 4.53 Gy plus V10 < 100.9 ml went into “all lower” group, and EDIC ≥ 4.53 Gy plus V10 ≥ 100.9 ml went into “all higher” group, and remaining went into “others” group; for NEU, EDIC < 5.79 Gy went into “lower” group, and EDIC ≥ 5.79 Gy went into “higher” group. Based on the multivariable analysis, “all lower” group in ALC (vs. “all higher” group; OR, 0.050; 95% CI, 0.010–0.251; *P* < 0.001), “all lower” group in WBC (vs. “all higher” group; OR, 0.177; 95% CI, 0.047–0.688; *P* = 0.011) and “lower” group in NEU (vs. “higher” group; OR, 0.401; 95% CI, 0.175–0.922; *P* = 0.031) were positively significant factors correlated with grade 3–4 cell toxicity during nCRT. And these associations were still significant after FDR adjustment. These results are shown in Table 3.

**Table 2:** The results of linear regression for the lowest cell count

	ALC			WBC			NEU		
	F	Adjusted R2 (SD)	*P* value	F	Adjusted R2 (SD)	*P* value	F	Adjusted R2 (SD)	*P* value
EDIC	28.710	0.209 (0.193)	< 0.001	8.587	0.067 (0.967)	0.004	5.441	0.041 (0.805)	0.022
V10	24.570	0.183 (0.196)	< 0.001	7.411	0.058 (0.972)	0.008	2.538	0.014 (0.836)	0.114
V20	22.471	0.170 (0.197)	< 0.001	6.114	0.046 (0.978)	0.015	2.094	0.010 (0.837)	0.151
V30	14.414	0.113 (0.204)	< 0.001	2.457	0.014 (0.994)	0.120	0.820	−0.002 (0.843)	0.367
V40	6.682	0.051 (0.211)	0.011	0.001	−0.010 (1.006)	0.977	0.194	−0.008 (0.845)	0.660
Sex (male vs. female)	3.877	0.027 (0.214)	0.052	1.761	0.007 (0.998)	0.187	3.147	0.020 (0.833)	0.079
Age (≤ 60 years vs. > 60 years)	0.171	−0.008 (0.218)	0.681	4.267	0.030 (0.986)	0.041	0.683	−0.003 (0.843)	0.410
Smoking history	3.588	0.024 (0.214)	0.061	0.788	−0.002 (1.003)	0.377	0.333	−0.006 (0.845)	0.565
Alcohol history	2.614	0.015 (0.215)	0.109	1.197	0.002 (1.000)	0.276	1.823	0.008 (0.839)	0.180
Tumor location (upper vs. others)	4.475	0.032 (0.213)	0.037	2.060	0.010 (0.996)	0.154	0.354	−0.006 (0.844)	0.553
GTV (≤ 48.4 cm^3^ vs. > 48.4 cm^3^)	1.522	0.005 (0.216)	0.220	1.525	0.005 (0.999)	0.220	0.408	−0.006 (0.844)	0.525
Clinical stage	1.842	0.008 (0.216)	0.178	1.502	0.005 (0.999)	0.223	0.003	−0.010 (0.846)	0.954
Chemotherapy regimen	0.195	−0.008 (0.218)	0.660	1.366	0.003 (1.000)	0.245	4.313	0.031 (0.829)	0.040

EDIC effective dose to immune cells; GTV, gross tumor volume

**Table 3:** The multivariate analysis for Grade 3–4 cell toxicity

	OR (95% CI)	*P* value	Q value
**ALC**			
Group		0.001	0.002
All lower (EDIC ≤ 4.63 Gy, V10 ≤ 140.3 ml)	0.050 (0.010–0.251)	< 0.001	0.001
Others	0.134 (0.025–0.726)	0.020	0.026
All higher (EDIC > 4.63 Gy, V10 > 140.3 ml)	Ref		
Tumor location			
Upper	0.912 (0.317–2.264)	0.864	0.864
Middle + Distal	Ref		
**WBC**			
Group		0.021	0.041
All lower (EDIC ≤ 4.53 Gy, V10 ≤ 100.9 ml)	0.177 (0.047–0.688)	0.011	0.041
Others	0.449 (0.165–1.225)	0.118	0.157
All higher (EDIC > 4.53 Gy, V10 > 100.9 ml)	Ref		
Age			
≤ 60 years	1.611 (0.681–3.810)	0.278	0.278
> 60 years	Ref		
**NEU**			
Group			
Lower (EDIC ≤ 5.79 Gy)	0.401 (0.175–0.922)	0.031	0.037
Higher (EDIC > 5.79 Gy)	Ref		
Chemotherapy regimen			
FP	0.191 (0.040–0.902)	0.037	0.037
TP	Ref		

ALC absolute lymphocyte count; WBC white blood cell count; NEU neutrophil count; EDIC effective dose to immune cells; Q values adjusted *P* values using Benjamini-Hochberg false discovery rate (FDR) correction for multiple hypothesis testing

### Kaplan-Meier analysis for HT between dosimetry subgroups

**Fig. 3:**
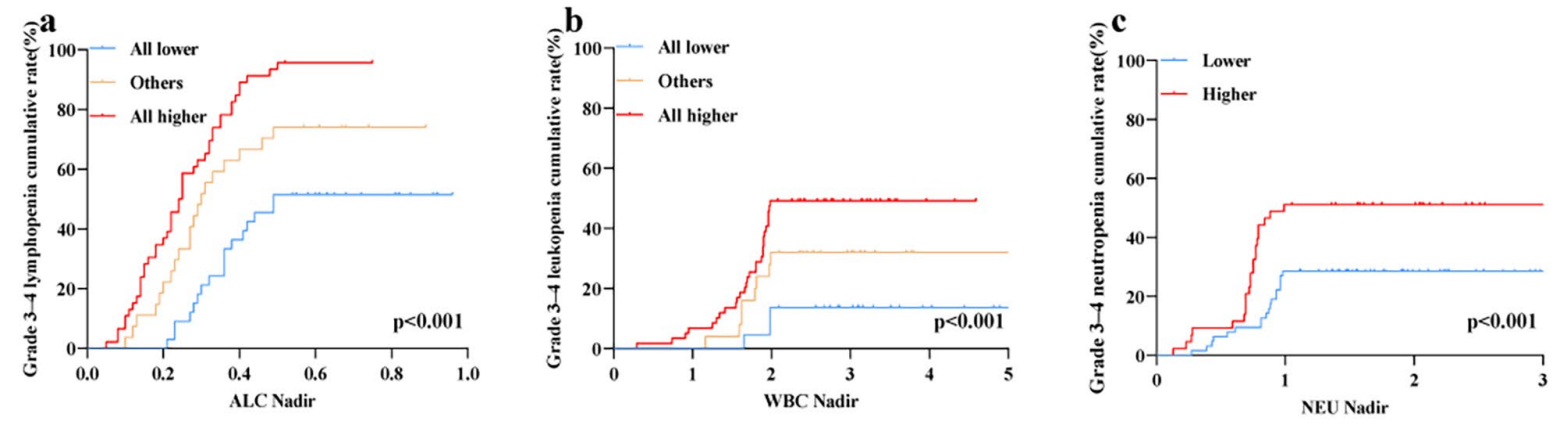
Kaplan–Meier analysis for grade 3–4 cell toxicity between dosimetry subgroups. **(a)** For grade 3–4 lymphopenia cumulative rate, *P* < 0.001; **(b)** for grade 3–4 leukopenia cumulative rate, *P* < 0.01; **(c)** for grade 3–4 neutropenia cumulative rate, *P* < 0.001

The Kaplan-Meier analysis showed that there was a significant difference between the three groups for grade 3–4 lymphopenia, leukopenia, and neutropenia, and all the *P*-values were < 0.001. The results are showen in Fig. 3.

## Discussion

In this study, we found that increasing vertebral body bone marrow radiation dose and EDIC were associated with the development of acute grade 3–4 HT in esophageal cancer patients treated with nCRT.

The relationships between vertebral body bone marrow radiation dosimetric parameters and HT in patients with ESCC during CRT has received increasing attention in recent years. Fabian [7] found in a study of patients with ESCC receiving CRT that increasing thoracic marrow radiation dose was associated with grade ≥ 3 HT. Liu et al [22] found in a study of 99 patients of thoracic oesophageal cancer treated with radical radiotherapy that the mean dose of the thoracic vertebrae and thoracic vertebrae V20 were associated with lymphocytopenia. Lee [23] analyzed 41 patients with esophageal cancer treated with nCRT and found that greater thoracic vertebrae dose such as thoracic vertebrae V10 and V20, were associated with increased grade ≥ 3 leukopenia risk. In addition to the effects of bone marrow irradiation dose, attention has been focused on the relationship between irradiation of the large number of vascular structures and blood pool in the thoracic cavity and hematological toxicity in recent years. Anderson [24] collected 46 patients with ESCC undergoing CRT and conducted multiple logistic regression analysis on the potential predictors of HT, finding that mean cardiopulmonary dose was associated with lower WBC and NEU nadirs (*p* < 0.05) and grade 4 lymphopenia was significantly associated with thoracic vertebral V10 and V20 (all *p* < 0.05). However, there may be some limitations to their use of the mean cardiopulmonary dose substitute for the peripheral hematological peripheral hematological dose, such as not taking into account the dose delivery and cardiovascular dynamics. Jin et al [14] further developed a model to estimate the EDIC, which is a surrogate for the estimated dose to the circulation immune pool, and discovered that greater EDIC was associated with a worse prognosis for patients with lung cancer in the Radiation Therapy Oncology Group (RTOG) 0617 trial. Higher EDIC (> 4.0 Gy) was also found to be strongly associated with severe lymphopenia in esophageal cancer patients treated with neoadjuvant or definitive concurrent chemoradiotherapy [15]. Tseng [16] analyzed the grade 4 lymphopenia in patients in the ESO-Shanghai 1 and ESO-Shanghai 2 trials and found that in ESCC patients who received definitive concurrent chemoradiotherapy, when the EDIC ≤ 8.3 Gy plus spleen V0.5 ≤ 11.1% and bone marrow V10 ≤ 33.2%, the incidence of grade 4 lymphopenia decreased. In our study, we reached some similar and some dissimilar conclusions as well.

In our study, we found that WBC, NEU, ALC, and PLT nadirs occurred at the 3–4 week of nCRT and then recovered, while HGB demonstrated a continuous decline throughout and ALC declined rapidly during nCRT, which is a reflection of cell‘s variation in intrinsic radiosensitivity and lymphocyte’s high radiosensitivity. And these results were simillar with a previous report by Anderson [24] et al. In analyzing the relationship between dosimetric parameters and HT, we combined EDIC and bone marrow dosimetric parameters into a single variable to eliminate the influence of possible multicollinearity between dosimetric parameters. This was supported by Tseng et al [16], where the authors demonstrated that smaller irradiations of the spleen and bone marrow and smaller EDIC reduced the risk of grade 4 lymphopenia, which echoes our finding. We found that increasing irradiation doses to thoracic bone marrow plus higher EDIC was associated with a risk of grade 3–4 lymphopenia and leukopenia, and higher EDIC was associated with a risk of grade 3–4 neutropenia, but not anaemia or thrombocytopenia. We found that increasing the irradiation dose to the bone marrow of the vertebral body was not associated with neutropenia, which may be due to the different radiosensitivity of neutrophils in the peripheral circulation and bone marrow. Berkow et al [25] found that morphologically mature neutrophils in the bone marrow exhibited reduced phagocytosis, slow stimulated superoxide anion production, reduced NADPH oxidase activity, and reduced AP activity compared to peripheral circulating neutrophils, which remained functionally immature. Kaplan-Meier analysis showed that the “all lower” group, which both had lower bone marrow irradiation dose and lower EDIC, had a lower incidence of grade 3–4 lymphopenia and leukopenia compared to the other two groups, and “lower group”, which had lower EDIC, had a lower incidence of grade 3–4 lymphopenia and neutropenia compared to the “higher” group. The results of the study suggest that adjusting the radiotherapy plans to reduce the irradiation of organs such as the vertebral bone marrow and the heart, lungs, and large blood vessels could reduce the risk of hematological toxicity, which may benefit the treatment of the tumor.

There are several limitations in our study. Our study is subject to a retrospective nature and may not be able to consider all potential factors, requiring further validation with prospective data. And we did not include the spleen in the analysis because the patients in our study all received very small incidental spleen irradiation or even none. It was found that even when patients with distal esophageal cancer received radiotherapy, not all patients received significant incidental radiation doses [26]. Further efforts should be made to improve it by taking these other organs into account in future studies.

## Conclusion

During neoadjuvant radiotherapy for ESCC, the dose of vertebral bone marrow irradiation and EDIC were significantly correlated with G3-4 leukopenia and lymphopenia, and EDIC was again significantly correlated with G3-4 neutropenia. The reduction of vertebral bone marrow irradiation and EDIC was effective in reducing the incidence of hematological toxicity.

## Data Availability

The datasets used and analysed during the current study are available from the corresponding author on reasonable request.

## Abbreviations

ALC: Absolute lymphocyte count
CBC: Complete blood counts
CTCAE: Common Terminology Criteria for Adverse Events
DVH: Dose volume histogram
EDIC: Effective dose to immune cells
ESCC: Esophageal squamous cell carcinoma
EUD: Equivalent uniform dose
GTV: Gross tumor volume
HGB: Hemoglobin
HT: Hematologic toxicity
IMRT: Intensity-modulated radiation therapy
ITD: Integral total dose
MBD: Mean body dose
MHD: Mean heart dose
MlD: Mean liver dose
MLD: Mean lung dose
MOD: Multiply mean organ dose
nCRT: Neoadjuvant chemoradiotherapy
NEU: Neutrophil count
PF: Cisplatin/fluorouracil
PLT: Platelet count
ROC: Receiver operating characteristic
RT: Radiation therapy
TP: Cisplatin/taxane
WBC: White blood cell count
